# BrECADD in three days: Implementation of a condensed intensive first‐line regimen in Hodgkin lymphoma

**DOI:** 10.1111/bjh.70771

**Published:** 2026-08-21

**Authors:** Christian Peter Jaworek, Maja Supprian, Paul Jan Bröckelmann, Hishan Tharmaseelan, Justin Ferdinandus, Dennis A. Eichenauer, Anne Sophie Robertz, Janina Jablonski, Vincent Combüchen, Jan‐Oliver Staak, Karin Töpelt, Jan‐Michel Heger, Jon Rischewski, Jannik Stemler, Wolfram Klapper, Peter Borchmann, Bastian von Tresckow

**Affiliations:** ^1^ Department I of Internal Medicine, Center for Integrated Oncology Aachen Bonn Cologne Duesseldorf (CIO ABCD), Faculty of Medicine and University Hospital of Cologne University of Cologne Cologne Germany; ^2^ German Hodgkin Study Group (GHSG) Cologne Germany; ^3^ MV Zentrum Hämatologie und Onkologie Cologne Germany; ^4^ Faculty of Medicine and University Hospital Cologne, Institute of Transfusion Medicine University of Cologne Cologne Germany; ^5^ Institute of Translational Research, Cologne Excellence Cluster on Cellular Stress Responses in Aging‐Associated Diseases (CECAD) Faculty of Medicine and University Hospital Cologne, University of Cologne Cologne Germany; ^6^ Department of Pathology, Hematopathology Section University Hospital Schleswig‐Holstein Campus Kiel Germany; ^7^ Department of Hematology and Stem Cell Transplantation West German Cancer Center, University Hospital Essen, University of Duisburg‐Essen Essen Germany

**Keywords:** BrECADD, brentuximab vedotin, first‐line treatment, Hodgkin lymphoma

## Abstract

Intensive multiagent treatment regimens have substantially improved outcomes in advanced‐stage classic Hodgkin lymphoma (cHL). Brentuximab vedotin (BV), etoposide, cyclophosphamide, doxorubicin, dacarbazine and dexamethasone (BrECADD) demonstrated the highest efficacy in cHL reported to date in a randomised phase III trial. Cytotoxic agents are conventionally administered during the first 4 days of a 21‐day cycle. A total of four cycles are applied to patients with a complete response after two cycles, otherwise six cycles. We developed a condensed BrECADD protocol, reducing intravenous administration days to only three (−25%). Between January 2024 and July 2026, 30 patients received 132 cycles of 3‐day BrECADD at our institution. Primary objective was to describe feasibility and safety; secondary objectives included response, progression‐free survival (PFS) and overall survival (OS). Grade ≥3 adverse events occurred in 23 patients (77%), predominantly haematological. Six patients (20%) had grade ≥3 infections (four viral, two bacterial) and six (20%) had pathogen‐unspecified febrile neutropenia. Twenty‐four patients (80%) required only four BrECADD cycles. There were no treatment discontinuations. After a median follow‐up of 18 months (range 4–29), 1‐year PFS and OS rates were both 100%. Based on these encouraging results, the condensed 3‐day protocol is considered feasible and we recommend it for future BrECADD use.

## Graphical abstract

BrECADD can be delivered over 3 days instead of four, reducing intravenous treatment days by 25% without compromising dose intensity. Among 30 patients with advanced‐stage Hodgkin lymphoma, no unexpected toxicity was observed; there were no early discontinuations, and 1‐year progression‐free and overall survival were both 100%.

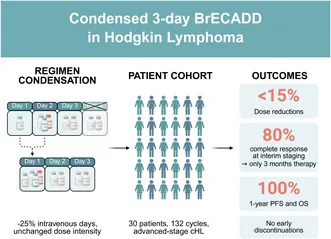

## INTRODUCTION

Classic Hodgkin lymphoma (cHL) is one of the most common malignancies in young adults and is highly curable with modern risk‐adapted therapy. The majority of patients present with advanced‐stage disease at diagnosis,^1^ requiring intensive systemic treatment to achieve durable remission.

Over the past decades, treatment strategies for advanced‐stage cHL have evolved considerably, balancing efficacy with long‐term toxicity. Numerous frontline regimens are currently used worldwide, including doxorubicin, bleomycin, vinblastine and dacarbazine (ABVD)‐based approaches,^2^ escalated bleomycin, etoposide, doxorubicin, cyclophosphamide, vincristine, procarbazine and prednisone (eBEACOPP),^3^, ^4^ antibody‐drug conjugate‐containing regimens,^5^, ^6^ and more recently, checkpoint inhibitor‐based combinations.^7^, ^8^ These strategies have achieved excellent outcomes, with long‐term progression‐free survival (PFS) and overall survival (OS) rates exceeding 80% according to many contemporary trials.^9^

Among these approaches, brentuximab vedotin (BV), etoposide, cyclophosphamide, doxorubicin, dacarbazine and dexamethasone (BrECADD) is the most effective first‐line therapy investigated in a randomised phase III trial for advanced‐stage cHL until now.^10^, ^11^ BrECADD is administered within a 21‐day cycle in a positron emission tomography (PET)‐guided manner. In the randomised phase III German Hodgkin Study Group (GHSG) HD21 trial, BrECADD demonstrated superior efficacy and a more favourable toxicity profile compared to eBEACOPP, establishing it as a new standard of care.^11^, ^12^, ^13^ BrECADD is conventionally administered as a 4‐day protocol.

In modern oncology, patient‐centred care and efficient use of healthcare resources are increasingly important. Multiday chemotherapy regimens impose substantial logistical burden on both patients and treatment centres, especially across repeated cycles. Reducing treatment days without compromising dose intensity enhances convenience, reduces hospital visits and improves workflow efficiency, addressing the growing concern of ‘time toxicity’, the considerable amount of time patients and their caregivers spend interacting with the healthcare system for cancer care, taken from their private, professional and social lives.^14^

To mitigate such practical considerations, we analysed a condensed 3‐day BrECADD protocol. In this modified protocol, cumulative doses and dose intensity remain unchanged, while the administration period is shortened by 1‐day per cycle. This approach is supported by prior phase III studies demonstrating the feasibility of administering BV concomitantly with combination chemotherapy on the same day, for example, the ECHELON‐1 trial for cHL (BV plus doxorubicin, vinblastine and dacarbazine; BV‐AVD)^5^ and the ECHELON‐2 trial for peripheral T‐cell lymphoma (BV plus cyclophosphamide, doxorubicin and prednisone; BV‐CHP).^15^

Here, we report the first experience with the condensed BrECADD regimen in patients with advanced‐stage cHL. The aim of this analysis was to describe feasibility and early clinical outcomes associated with a 3‐day BrECADD regimen in routine clinical practice at the University Hospital of Cologne.

## METHODS

### Protocol design

Following an institutional practice change in January 2024, all patients considered eligible for BrECADD were treated with a newly developed condensed protocol in routine clinical care (Table S1).

In the standard 4‐day BrECADD regimen, BV is given as single agent on day 1, followed by combination chemotherapy on days 2–4 and oral dexamethasone on days 2–5 (Figure 1A). In the condensed 3‐day BrECADD regimen, BV is given concomitantly with the first day of combination chemotherapy, shortening the intravenous (iv) administration period to days 1–3 with oral dexamethasone on days 1–4 (Figure 1B; Figure S1). Cumulative doses and dose intensity remain unchanged. Granulocyte‐colony stimulating factor (G‐CSF) was administered as primary prophylaxis. Importantly, predefined de‐escalation strategies and dose‐level reduction criteria remained identical to the HD21 protocol (Figure 1C, Table S2). The day‐by‐day administration schedule including supportive care (Table S3) and the full dose‐reduction algorithm (Table S4) follow the HD21 protocol. The number of treatment cycles was determined by PET according to the HD21 protocol, with interim PET performed after 2 cycles. Response was assessed using the Deauville 5‐point scale, with scores 1–3 defined as complete response (CR).

**FIGURE 1:**
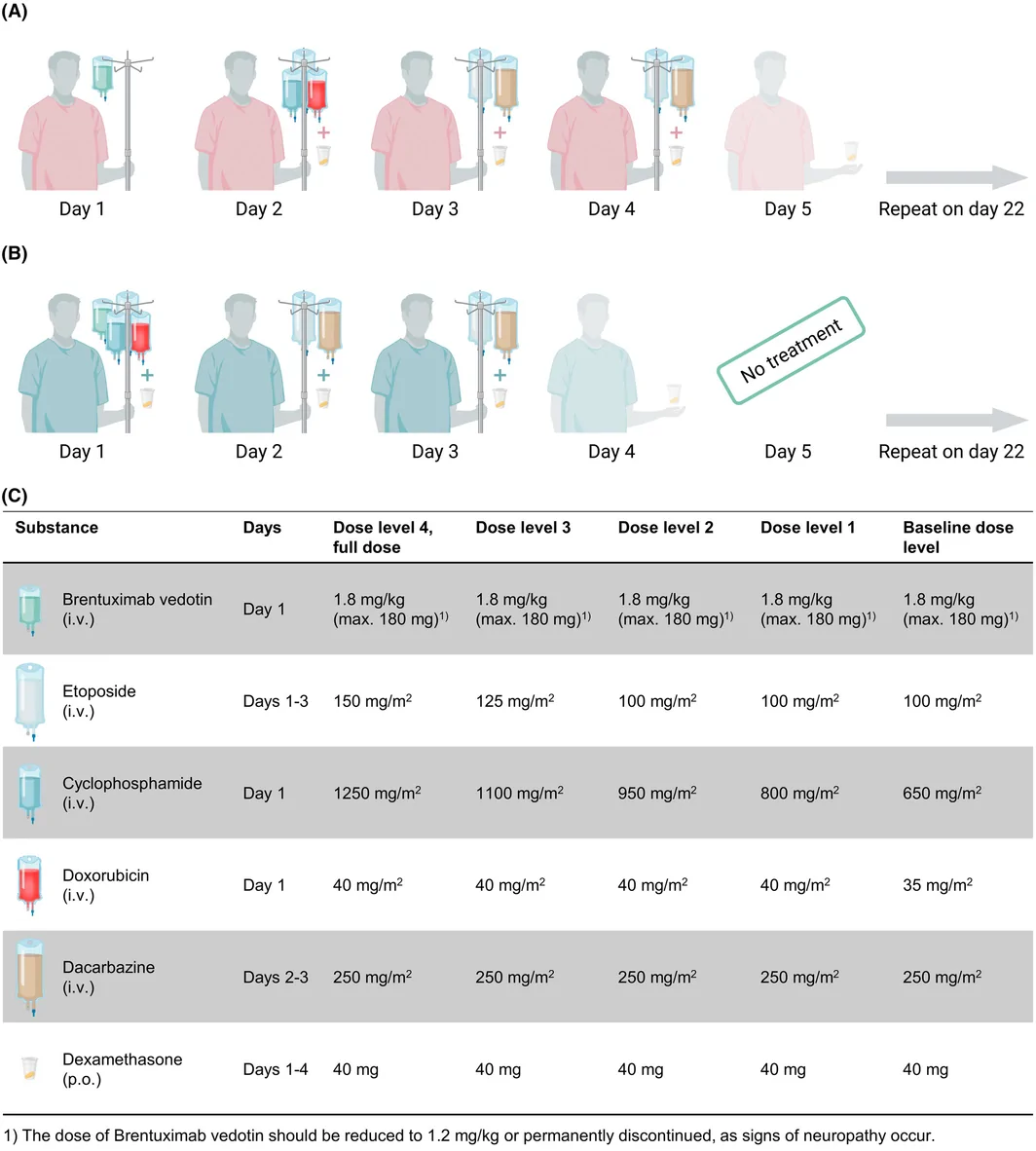
(A) Conventional 4‐day regimen as published in the HD21 trial; (B) new 3‐day regimen; (C) administration days and dose levels of both regimens.

### Patients

Patients aged 18–75 years with advanced‐stage cHL were eligible. Advanced stage was defined as Ann Arbor stages III/IV as well as stage II with B‐symptoms and one or both risk factors large mediastinal mass (≥a third of the maximum thoracic diameter) or extra‐nodal involvement. Eligibility further required the absence of prior systemic anti‐cancer therapy, with the exception of a corticosteroid pre‐phase.

### Data collection and statistics

We retrospectively collected clinical data of eligible patients treated from 10 January 2024 to 17 July 2026 at the University Hospital of Cologne and received the condensed BrECADD protocol. At baseline, this comprised patient demographics, including age at diagnosis, sex and disease characteristics including histological subtype, Eastern Cooperative Oncology Group (ECOG) performance status, presence of B‐symptoms, Ann Arbor stage^16^ and risk factors as defined by the GHSG.^17^

Treatment‐related data were systematically collected for each treatment cycle and included cycle start dates, planned and administered dose levels and dose reductions or omissions. In cases of single‐ or multisubstance dose deviations, the underlying reasons were extracted. In addition, data on unplanned hospitalisations, number of required red blood cell and platelet transfusions, PET‐based response assessments, total number of administered cycles and the use of consolidation radiotherapy were documented.

Adverse events grade ≥3 according to Common Terminology Criteria for Adverse Events (CTCAE) version 5.0^18^ recorded during and after treatment were analysed. For haematological events, this comprised anaemia with haemoglobin <8 g/dL, thrombocytopenia with platelet counts <50 × 10^9^/L, and leucopenia with leucocyte counts <2 × 10^9^/L. Lower‐grade adverse events were not captured due to limited attributional certainty. Descriptive statistics were performed for patient characteristics, treatment outcomes and adverse events. PFS and OS were assessed as clinical outcomes. PFS was defined as time from diagnosis until disease progression, relapse or death from any cause or censored at the date of last physician contact. OS was defined as time from diagnosis to death from any cause. Both were estimated using Kaplan–Meier method. All included patients had complete clinical documentation with no missing data. The data collection cut‐off was 17 July 2026. Patients with ongoing treatment at the cut‐off were not included.

## RESULTS

### Patients

Of 41 patients treated with the condensed BrECADD protocol, five patients switched treatment sites (12%), two patients did not receive all cycles per condensed schedule (5%), two patients received BrECADD as salvage therapy (5%), one patient received prior pembrolizumab (2%) and one patient was lost to follow‐up during therapy (2%). In the final analysis, 30 patients with advanced‐stage cHL were included (Figure S2). The median age at diagnosis was 31 years (range 18–62 years), with two‐thirds of patients aged between 20 and 39 years (*n* = 20, 67%). The cohort comprised 11 (37%) female and 19 (63%) male patients. At baseline, 11 patients (37%) had Ann Arbor stage IIB disease, while 12 (40%) presented with stage III (IIIA *n* = 4, 13%; IIIB *n* = 8, 27%) and 7 (23%) with stage IV disease (IVA *n* = 1, 3%; IVB *n* = 6, 20%). ECOG performance status was 0 in 23 patients (77%), 1 in 5 patients (17%), 2 in 1 patient (3%) and 3 in 1 patient (3%). Clinical risk factors according to GHSG were present in all patients, with involvement of ≥3 areas in 28 patients (93%), large mediastinal mass in 17 patients (57%), elevated erythrocyte sedimentation rate in 15 patients (50%), and extra‐nodal involvement in 13 (43%) patients. The predominant histological subtype was nodular sclerosis (*n* = 17, 57%), followed by mixed cellularity (*n* = 7, 23%) and lymphocyte depleted (n = 1, 3%), while 5 cases (17%) were classified as cHL without further subtyping. Reference pathology was performed in 25 patients (83%) (Table 1).

**TABLE 1:** Patient and disease characteristics.

Three‐day BrECADD population	*N* = 30
Age	
Median (range), years	31 (18–62)
Distribution, no. (%)	
18–19	2 (7%)
20–29	10 (33%)
30–39	10 (33%)
40–49	5 (17%)
50–59	2 (7%)
60–69	1 (3%)
Sex	
Female	11 (37%)
Male	19 (63%)
Ann Arbor stage	
IIB	11 (37%)
IIIA	4 (13%)
IIIB	8 (27%)
IVA	1 (3%)
IVB	6 (20%)
ECOG performance status	
0	23 (77%)
1	5 (17%)
2	1 (3%)
3	1 (3%)
GHSG risk factors	
Involvement of ≥3 areas	28 (93%)
Elevated ESR	15 (50%)
Large mediastinal mass	17 (57%)
Extra‐nodal involvement	13 (43%)
Histological subtype	
Nodular sclerosis	17 (57%)
Mixed cellularity	7 (23%)
Classic HL without subtyping	5 (17%)
Lymphocyte depleted	1 (3%)
Reference pathology	
Performed, no (%)	25 (83%)

*Note*: Data are *n* (%), median (range) or *n*/*N* (%).

Abbreviations: BrECADD, brentuximab vedotin, etoposide, cyclophosphamide, doxorubicin, dacarbazine, and dexamethasone; ECOG, Eastern Cooperative Oncology Group; ESR, erythrocyte sedimentation rate; HL, Hodgkin lymphoma.

### Feasibility

Across the 30 analysed patients receiving the condensed 3‐day BrECADD protocol, a total of 132 treatment cycles were administered. No patient switched treatment and no early treatment discontinuations occurred. No relevant treatment deviations were observed throughout all administered cycles apart from predefined dose‐level adjustments and BV dose modifications where clinically indicated.

The observed median cycle interval was 21 days (range 19–31 days). Regarding treatment setting, 89 cycles (67%) were administered in an outpatient setting and 43 cycles (33%) in an inpatient setting. Most cycles were delivered at dose level 4 (full dose), accounting for 118 cycles (89%), whereas 12 cycles (9%) were administered at dose level 3, and 1 cycle each at dose level 2 (1%) and baseline dose level (1%). Overall, three patients (10%) received at least one red blood cell transfusion (median 2, range 1–3), and six patients (20%) received at least one platelet transfusion (median 1, range 1–2). Unplanned hospitalisation occurred in 11 patients (37%). BV was administered at the full dose of 1.8 mg/kg in 127 cycles (96%). One cycle (1%) was given with a reduced BV dose of 1.2 mg/kg due to grade 2 peripheral sensory neuropathy. BV was omitted in 4 cycles (3%), all in one patient with pre‐existing severe peripheral sensory and motor neuropathy prior to treatment initiation. No unplanned dose reductions and no omissions were recorded for the other components of the protocol (Table 2).

**TABLE 2:** Therapy outcomes.

Therapy outcomes per patient	*N* = 30 patients
Response after 2 cycles, no (%)	
Complete remission	24 (80%)
Partial remission	6 (20%)
Total treatment cycles, no (%)	
4 cycles	24 (80%)
6 cycles	6 (20%)
Consolidative radiation for PET‐positive residual disease, no (%)	2 (7%)
Unplanned hospitalisation	
Patients with unplanned hospitalisation, no (%)	11 (37%)
Days of unplanned hospitalisation per patient, median (range)	6 (1–16)
Blood transfusions	
Patients with red blood cell transfusions, no (%)	3 (10%)
Number of red blood cell transfusions per patient, median (range)	2 (1–3)
Patients with platelet transfusions, no (%)	6 (20%)
Number of platelet transfusions per patient, median (range)	1 (1–2)

Therapy outcomes per cycle	*N* = 132 cycles
Cycle interval	
Expected length, days	21
Actual length, median (range)	21 (19–31)
Healthcare facility	
Outpatient	89 (67%)
Inpatient	43 (33%)
Dose levels	
Dose level 4 (full dose)	118 (89%)
Dose level 3	12 (9%)
Dose level 2	1 (1%)
Baseline dose level	1 (1%)
Brentuximab vedotin doses	
Full dose (1.8 mg/kg)	127 (96%)
Reduced dose (1.2 mg/kg)	1 (1%)
Not administered	4 (3%)
ECADD single substance doses	
No deviation, no (%)	132 (100%)

*Note*: Data are *n* (%), median (range) or *n*/*N* (%).

Abbreviations: ECADD, etoposide, cyclophosphamide, doxorubicin, dacarbazine and dexamethasone; PET, positron emission tomography.

### Safety

Haematological adverse events CTCAE grade ≥3 occurred in 23 patients (77%). Leucopenia was observed in 22 patients (73%), thrombocytopenia in 14 patients (47%) and anaemia in 8 patients (27%). Neutropenic fever occurred in six patients (20%), and grade ≥3 infections occurred in six patients (20%). Of the six patients with grade ≥3 infections, four had viral infections (respiratory syncytial virus, coronavirus disease 2019, two with influenza A) and two bacterial bloodstream infections (*Staphylococcus epidermidis*, *Klebsiella*). The *S. epidermidis* case occurred without prior antibiotic prophylaxis, whereas the *Klebsiella* isolate was cotrimoxazole‐resistant following prior prophylaxis.

Cardiac disorders grade ≥3 occurred in three patients (10%), whereas nervous system events grade ≥3 were reported in two patients (7%) and gastrointestinal events grade ≥3 in one patient (3%). No grade ≥3 hepatobiliary, renal and urinary, respiratory, thoracic or mediastinal, skin and subcutaneous tissue or drug fever events were observed. Likewise, no grade ≥3 allergic reactions or avascular necrosis were documented.

Particular attention was further given to toxicities potentially associated with BV. One patient experienced grade 2 peripheral sensory neuropathy; no grade ≥3 peripheral sensory neuropathy or peripheral motor neuropathy was observed. There were also no infusion‐related reactions (Table 3).

**TABLE 3:** Severe adverse events during therapy.

Adverse events ≥CTCAE grade 3	*N* = 23/30
Leucopenia	22 (73%)
Thrombocytopenia	14 (47%)
Anaemia	8 (27%)
Infection	6 (20%)
Neutropenic fever	6 (20%)
Cardiac disorders	3 (10%)
Nervous system disorder (other than neuropathy)	2 (7%)
Gastrointestinal disorders	1 (3%)
Hepatobiliary disorders	0 (0%)
Peripheral sensory neuropathy	0 (0%)
Peripheral motor neuropathy	0 (0%)
Renal and urinary disorders	0 (0%)
Respiratory, thoracic and mediastinal disorders	0 (0%)
Skin and subcutaneous tissue disorders	0 (0%)
Drug fever	0 (0%)
Allergy	0 (0%)
Avascular necrosis	0 (0%)
Infusion‐related reaction	0 (0%)

### Efficacy

Interim response assessment after two cycles showed a response rate of 100%, with a CR in 24 patients (80%) and a partial response (PR) in 6 patients (20%). All patients with interim CR proceeded with a reduced‐length treatment consisting of four total cycles.

At the end of systemic treatment, 28 patients (93%) achieved a PET‐negative CR (Deauville 1–3), whereas 2 patients (7%) had PET‐positive residual findings (Deauville 4–5). The latter patients received consolidation radiotherapy. Radiotherapy was only administered in those 2 patients.

During follow‐up, no cases of disease progression or relapse were recorded, and no deaths occurred. The 1‐year PFS and OS rates were both 100%. Median follow‐up was 18 months (range 4–29) (Table 2, Figure 2).

**FIGURE 2:**
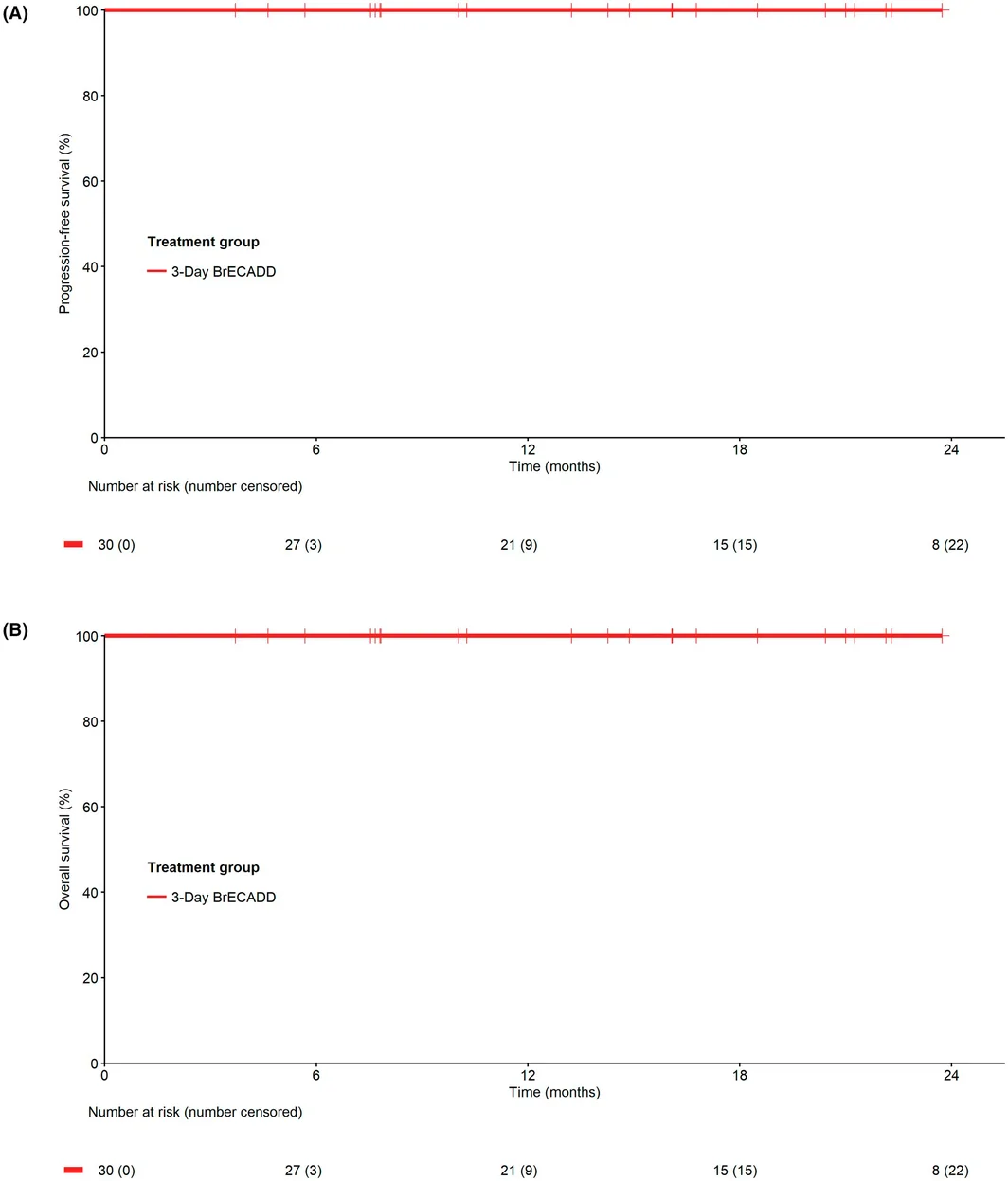
(A) Progression‐free survival (24 months); (B) Overall survival (24 months).

## DISCUSSION

The following major findings were observed in our analysis: (1) The 3‐day BrECADD regimen proved feasible and supports implementation in routine clinical practice. (2) No concerning toxicity signals were observed with no progression, relapse or death observed at a median follow‐up of 18 months. (3) Treatment response was favourable, with a CR at interim PET in 80% of patients (HD21: 64%), allowing most patients to complete treatment within approximately 3 months.

Treatment was delivered largely according to the intended BrECADD schedule, with a median cycle duration of 21 days, most cycles administered at full dose level, and no relevant dose reductions or omissions outside the predefined dose‐adjustment algorithm. Importantly, no early treatment discontinuations occurred, indicating that shortening the administration schedule from four to 3 days did not compromise treatment delivery. Of note, four of five patients could be managed with the shortened treatment approach of only four cycles. The safety results further support the feasibility of this approach, as no unexpected toxicity signals were observed. There was one case of grade 2 neurotoxicity but no higher grade neurotoxicity or infusion‐related reactions associated with BV. Although follow‐up duration is still limited, the clinical outcomes were encouraging, with no progression, relapse or death observed and 1‐year PFS and OS rates of 100%.

The lower rate of interim PET‐positive patients than in HD21 is most plausibly explained by chance in this small cohort, as a relation to the condensed schedule appears unlikely with cumulative doses and dose intensity unchanged. Growing institutional experience with treatment delivery, supportive care and interim PET assessment since the conduct of HD21 may nevertheless have contributed.

A major advantage of the 3‐day BrECADD regimen is the reduction in healthcare contacts. Compared with the conventional 4‐day schedule, condensing treatment to 3 days reduces chemotherapy treatment days by up to 25%, optimising healthcare resource utilisation.^19^, ^20^ This has significant relevance for both patients and treatment centres, as fewer treatment days mean less logistical burden, fewer scheduling demands and lower pressure on outpatient and inpatient resources. In addition, the shorter schedule may reduce conflicts with weekends and public holidays, thereby facilitating timely treatment delivery in routine care.

From the patient perspective, fewer treatment days may also reduce the overall time spent on all aspects of treatment delivery, such as travelling and time in waiting rooms, sometimes referred to as time toxicity^21^ and financial toxicity,^20^ the direct and indirect costs of cancer care borne by patients. These practical benefits are particularly relevant when comparing BrECADD with alternative first‐line strategies. In our cohort, most patients completed therapy within approximately 3 months, as 80% needed only four cycles of therapy. By contrast, programmed cell death protein 1 (PD1) inhibitor‐based regimens such as Nivo‐AVD (nivolumab, doxorubicin, vinblastine, dacarbazine)^7^ are typically administered over approximately 6 months, effectively doubling the overall treatment duration. While PD1‐based approaches have shown promising efficacy, they are also associated with immune‐related adverse events that may affect multiple organ systems and occasionally lead to long‐term morbidity.^22^, ^23^, ^24^ In contrast, BrECADD is a time‐limited cytotoxic regimen with a well‐characterised and predictable toxicity profile and no risk of chronic immune‐mediated toxicity.^25^ Moreover, outcomes after relapse remain limited despite contemporary salvage strategies,^26^ and anti‐PD1 antibodies represent an effective salvage option that may be forfeited by their use in first line,^27^ underlining the importance of maximising cure rates with initial treatment. The 3‐day BrECADD regimen may therefore represent an attractive strategy combining high efficacy, short treatment duration and improved practical deliverability.

However, our results might not be fully representative as this single‐centre analysis included a rather small number of patients. Statistical analysis is descriptive, with no formal hypothesis testing. The study was conducted at a specialised lymphoma centre with extensive experience from the HD21 trial and with structured supportive care like risk‐adapted antibiotic prophylaxis,^28^ consistent G‐CSF support, expert pathology review^29^ and high experience in PET‐based response assessment. These factors may have contributed to the low adverse event rate and favourable treatment outcomes. Generalizability may therefore be restricted. As duration of follow‐up remains limited, no conclusions on long‐term disease control and late toxicity can be drawn.

In conclusion, this analysis for the first time provides data with a condensed 3‐day BrECADD regimen in advanced‐stage cHL. Beyond evaluating the modified schedule itself, the analysis also provides real‐world evidence on the use of BrECADD in routine clinical practice, showing favourable treatment delivery and response outcomes. Taken together, these findings support the implementation of the 3‐day BrECADD regimen as an attractive administration schedule.

## AUTHOR CONTRIBUTIONS

**Christian Peter Jaworek:** Writing – original draft; writing – review and editing; data curation; investigation. **Maja Supprian:** Writing – review and editing; formal analysis; writing – original draft; data curation. **Paul Jan Bröckelmann:** Writing – review and editing; investigation. **Dennis A. Eichenauer:** Investigation; writing – review and editing. **Jan‐Michel Heger:** Investigation; writing – review and editing. **Hishan Tharmaseelan:** Writing – review and editing; investigation. **Karin Töpelt:** Investigation; writing – review and editing; data curation. **Bastian von Tresckow:** Conceptualization; investigation; writing – review and editing; writing – original draft; project administration; validation. **Justin Ferdinandus:** Investigation; writing – review and editing. **Peter Borchmann:** Conceptualization; investigation; writing – review and editing; project administration. **Janina Jablonski:** Formal analysis; writing – review and editing. **Jon Rischewski:** Investigation; writing – review and editing. **Jan‐Oliver Staak:** Data curation; writing – review and editing; investigation. **Vincent Combüchen:** Data curation; writing – review and editing. **Jannik Stemler:** Writing – review and editing; investigation. **Wolfram Klapper:** Writing – review and editing; data curation. **Anne Sophie Robertz:** Investigation; writing – review and editing.

## FUNDING INFORMATION

This research received no external funding.

## CONFLICT OF INTEREST STATEMENT

PJB is an advisor or consultant for Hexal, Merck Sharp & Dohme (MSD), Need AI, Stemline, Takeda and Therakos; holds stock options in Need AI; has received honoraria from AstraZeneca, BeOne Medicines, Bristol‐Myers Squibb/Celgene (BMS), Janssen‐Cilag, Lilly, MSD, Need AI, Roche, Stemline and Takeda; and reports research funding from BeOne Medicines (institution), BMS (institution), MSD (institution) and Takeda (institution). JF is an advisor or consultant for Pfizer and Takeda and has received honoraria from Takeda. JMH reports research funding from Incyte, MorphoSys and Novartis; has received honoraria or is an advisor or consultant for Incyte, Roche, Novartis, Miltenyi Biotec, Sobi and SERB Pharmaceuticals; has received travel support from Sobi and SERB Pharmaceuticals; and is a member of ASH, EHA, DGHO, DGIIN and GLA. JS has received research grants from the German Federal Ministry of Education and Research, the Medical Faculty of the University of Cologne, Basilea Pharmaceutica, Noscendo and Scynexis; has received speaker honoraria from AbbVie, Akademie für Infektionsmedizin, Forum für Medizinische Fortbildung, Hikma, Gilead Kite, Lilly, Mundipharma, Pfizer and VITIS; has been a consultant or participated on advisory boards for AiCuris, Basilea, Gilead Kite, Mundipharma, Shionogi, Alvea Vax and Micron Research; and has received travel support from Page Medical, Meta Alexander Foundation, German Society for Infectious Diseases and Shionogi; all outside the submitted work. PB is an advisor or consultant for Takeda, BMS, Roche, Amgen, Novartis, Miltenyi Biotec, Gilead Kite, MSD, Incyte and BeOne Medicines; has received honoraria from Takeda, Novartis, BMS, Roche, MSD, Celgene, Miltenyi Biotec, Gilead Kite and AbbVie; and reports research funding from Takeda, MSD, Incyte and Amgen. BvT is an advisor or consultant for AbbVie, Allogene, Amgen, BMS, Cerus, Gilead Kite, Incyte, IQVIA, Janssen‐Cilag, Lilly, MSD, Miltenyi Biotec, Novartis, Noscendo, Pentixapharm, Pfizer, Pierre Fabre, Qualworld, Regeneron, Roche, SERB Pharmaceuticals, Sobi and Takeda; has received honoraria from AbbVie, AstraZeneca, BMS, Gilead Kite, Incyte, Janssen‐Cilag, Lilly, MSD, Novartis, Roche, SERB Pharmaceuticals and Takeda; reports research funding from Esteve (institution), MSD (institution), Novartis (institution) and Takeda (institution); reports travel support from AbbVie, AstraZeneca, Gilead Kite, Janssen‐Cilag, Lilly, MSD, Pierre Fabre, Roche, Takeda and Novartis; and is a member of steering committees for Regeneron (institution) and Takeda. CPJ, MS, HT, DAE, ASR, JJ, VC, JOS, KT, JR and WK do not declare any potential conflicts of interest.

## Supporting information


**Appendix S1:** BrECADD 3‐day protocol template.
**Table S1:** General treatment information.
**Figure S1:** Therapy course overview.
**Table S2:** Substance doses and dose levels.
**Table S3:** Day‐by‐day administration.
**Table S4:** Treatment management.
**Appendix S2:** Supplemental material.
**Figure S2:** CONSORT chart.

## Data Availability

The data that support the findings of this study are available on request from the corresponding author. The data are not publicly available due to privacy or ethical restrictions.
