# Supplementary material for: BrECADD in three days: Implementation of a condensed intensive first‐line regimen in Hodgkin lymphoma
Source: Br J Haematol. 2026 Aug 21;209(5):2094–102. doi: 10.1111/bjh.70771 (PMC13748422; doi:10.1111/bjh.70771)
Supplement: Supplementary file 1 [file BJH-209-2094-s001.docx]

**Appendix 1: BrECADD 3-day protocol template**

For classic Hodgkin Lymphoma (cHL) | 3-day administration schedule

**Template use and responsibility**

This appendix is intended as a protocol template for local adaptation. It is not a stand-alone chemotherapy prescription. Total drug doses, supportive medication, laboratory thresholds, contraindications, infusion practice, and monitoring requirements must be reviewed and approved according to local institutional policies, national guidance, product information, and the treating physician's judgement before clinical use. **Responsibility for administration remains with the user.**

**Table S1: General treatment information**

| **Field** | **Content** |
| --- | --- |
| Treatment regimen | Condensed BrECADD regimen, full dose level 4, adapted from GHSG HD21 trial (Borchmann et al., Lancet 2024) |
| Cycle length | Repeat every 22 days, if clinical and laboratory criteria for treatment are met |

**Figure S1: Therapy course overview**

**Table S2: Substance doses and dose levels**

1) The dose of Brentuximab vedotin should be reduced to 1.2 mg/kg or permanently discontinued, as signs of neuropathy occur (see SmPC).

**Table S3: Day-by-day administration**

| **Day** | **Time** | **Purpose** | **Therapy (dose level 4, full dose)** | **Notes** |
| --- | --- | --- | --- | --- |
| Day 1 | 08:00 – 09:00 | Hydration | Balanced crystalloid solution 500 ml i.v. over 1 h |  |
| Day 1 | 08:00 | Anti-infective prophylaxis | Cotrimoxazole 960 mg tablets | From day 1 every Mon, Wed, Fri, throughout the entire treatment course until 1 month after last BrECADD application |
| Day 1 | 08:00  and  20:00 | Anti-infective prophylaxis | Valaciclovir 500 mg tablets | Non-mandatory but recommended: 1-0-1 from day 1 throughout the entire treatment course until 1 month after last BrECADD application |
| Day 1 | 08:00 | Tumor lysis prophylaxis | Allopurinol 300 mg tablets | If clinically indicated |
| Day 1 | 08:00 | Antiemetic prophylaxis | Aprepitant 125 mg tablets |  |
| Day 1 | 08:00 | Antiemetic prophylaxis | Granisetron 1 mg i.v. | Total volume 1 ml  Alternatively Ondansetron 8 mg i.v., observe maximum daily dose |
| Day 1 | 08:00 |  | **Dexamethasone 40 mg tablets** |  |
| Day 1 | 08:00 – 08:30 |  | **Brentuximab vedotin 1.8 mg/kg in 250 ml NaCl 0.9% i.v. over 30 min** | Maximum 180 mg |
| Day 1 | 08:30 | Uroprotection | Mesna 400 mg bolus | Total volume 4 ml  Administer before cyclophosphamide |
| Day 1 | 08:30 – 10:00 | Uroprotection | Mesna 800 mg/m² BSA plus KCl 20 mmol in 1000 ml NaCl 0.9% | Accompanying infusion with cyclophosphamide / doxorubicin |
| Day 1 | 08:30 – 9:30 |  | **Cyclophosphamide 1250 mg/m^2^ BSA in 500 ml NaCl 0.9% i.v. over 1 h** |  |
| Day 1 | 09:30 – 10:00 |  | **Doxorubicin 40 mg/m² BSA in 500 ml NaCl 0.9% i.v. over 30 min** |  |
| Day 1 | 10:00 – 11:00 |  | **Etoposide 150 mg/m² BSA in 1000 ml NaCl 0.9% i.v. over 1 h** | Alternatively Etoposide phosphate |
| Day 2 | 08:00 – 10:00 | Hydration | Balanced crystalloid solution 1000 ml i.v. over 2 h |  |
| Day 2 | 08:00 | Antiemetic prophylaxis | Aprepitant 80 mg tablets |  |
| Day 2 | 08:00 | Antiemetic prophylaxis | Granisetron 1 mg i.v. | Total volume 1 ml  Alternatively Ondansetron 8 mg i.v., observe maximum daily dose |
| Day 2 | 08:00 |  | **Dexamethasone 40 mg tablets** |  |
| Day 2 | 08:00 – 10:00 |  | **Dacarbazine 250 mg/m² BSA in 250 ml NaCl 0.9% over 2 h** |  |
| Day 2 | 10:00 – 11:00 |  | **Etoposide 150 mg/m² BSA in 1000 ml NaCl 0.9% i.v. over 1 h** | Alternatively Etoposide phosphate |
| Day 3 | 08:00 – 10:00 | Hydration | Balanced crystalloid solution 1000 ml i.v. over 2 h |  |
| Day 3 | 08:00 | Antiemetic prophylaxis | Aprepitant 80 mg tablets |  |
| Day 3 | 08:00 | Antiemetic prophylaxis | Granisetron 1 mg i.v. | Total volume 1 ml  Alternatively Ondansetron 8 mg i.v., observe maximum daily dose |
| Day 3 | 08:00 |  | **Dexamethasone 40 mg tablets** |  |
| Day 3 | 08:00 – 10:00 |  | **Dacarbazine 250 mg/m² BSA in 250 ml NaCl 0.9% over 2 h** |  |
| Day 3 | 10:00 – 11:00 |  | **Etoposide 150 mg/m² BSA in 1000 ml NaCl 0.9% i.v. over 1 h** | Alternatively Etoposide phosphate |
| Day 4 | 08:00 |  | **Dexamethasone 40 mg tablets** |  |
| Day 4 onward | 08:00 | G-CSF support | Pegfilgastrim 6 mg pre-filled syringe s.c.  Alternatively  G-CSF 150 µg/m² s.c. daily at 08:00 until leukocyte count is > 1 000/mm³ on three consecutive days | Mandatory! |
| Day 8 onward | 08:00  and  20:00 | Anti-infective prophylaxis | Ciprofloxacin 250 mg tablets | 1-0-1 until end of cytopenia (recovery of neutrophil granulocytes (ANC) > 500/mm³ or leukocytes > 1 000/mm³) |

**Table S4: Treatment management**

| **Field** | **Content** |
| --- | --- |
| Pre-treatment phase | A pre-treatment phase with 40 mg dexamethasone over 4 days may be administered if clinically indicated. |
| Continuation of subsequent cycle | Prerequisite on day 22 (first day of subsequent cycle): **leukocytes > 2 500/mm³ or** **neutrophil granulocytes (ANC) > 1 500/mm³** and **thrombocytes >80 000/mm³**, no active infection or severe toxicity. Otherwise, therapy postponement or dose reduction according to respective criteria. Also with leukocytes > 15 000/mm³ with G-CSF therapy, postpone until values are lower again. |
| Dose reduction | Dose reduction follows a predefined de-escalation strategy, which is based upon the occurrence of toxic events in previous cycles. The following are regarded as toxic events:   - Leukopenia for more than 4 days (leukocytes < 1 000/mm³) - Thrombocytopenia on one or more days (thrombocytes < 25 000/mm³) - Infection CTCAE grade 4 - Other CTCAE grade 4 toxicities, e.g. mucositis - Treatment delay of more than 2 weeks due to inadequate recovery of blood values   If one or more toxic events occur in a given cycle, the dose in all following cycles has to be reduced by one dose level. If toxic events occur in two successive cycles, the doses are reduced to baseline level. (For dose levels, see page 1) |
| Additional checks | Blood count **twice a week**, check differential blood counts 2-3 times on the expected nadir on **days 8-15**, also check creatinine, urea, uric acid, GOT, GPT, gamma-GT, AP, bilirubin, potassium, sodium and urine status. |
|  |  |
| **PET-guided treatment for advanced stage cHL** | |
| Number of cycles | Response-adapted treatment with 4 or 6 cycles, with interim PET assessment after 2 cycles |
| Interim PET/CT timepoint and evaluation | PET-2 including diagnostic CT on days 17-21 of the second cycle. Patients negative PET-2 receive 4 cycles in total, Patients positive PET-2 receive 6 cycles in total. |
| Final PET/CT timepoint and evaluation | PET-4 / PET-6 within 4 weeks after last therapy cycle. Only if this PET-4 or -6 is positive, residual tumors are irradiated with 30 Gy. |
| Response assessment | A Deauville score of 1-3 is defined as PET-negative, a Deauville score of 4-5 as PET-positive |

**Drug-specific notes**

**Brentuximab vedotin**

Side effects: peripheral polyneuropathy (common), haematotoxicity (dose adjustment may be necessary), tumor lysis syndrome, anaphylaxis (rare).

Infusion rate at start 30 ml/h. Increase rapidly, if well tolerated.

**Cyclophosphamide**

Caution: Adjust dose according to renal function (for GFR < 60 ml/min, 75% of the dose). Ensure adequate fluid intake (drinking, irrigation, urine output should be at least 200 ml/h).

Side effects: Hemorrhagic cystitis. Mesna prophylaxis at 500 – 1 000 mg/m². Premedication 800 mg absolute as bolus without obligatory Mesna administration in irrigation, for cyclophosphamide > 1,000 mg/m² 20% of the dose equivalent should be administered as bolus and 80% in irrigation before therapy. Possible cardiotoxicity at high doses, pneumonitis, neurotoxicity.

**Doxorubicin (Adriamycin)**

Caution: Threshold dose for cardiotoxicity 550 mg/m² (cardiac and individual risk assessment at least by means of cardiac echo). Dose reduction for bilirubin levels > 1.5 mg/dl. Administer in light protection.

Caution: Must be administered with irrigation!

**Etoposide**

Side effects: Blood pressure drop (rare), arrhythmias (infusion duration at least 1 hour). Reduce dose to 75% if GFR < 60 ml/min; for bilirubin > 1.5 mg/dl.

Caution: Calcium antagonists may increase toxicity, do not administer etoposide and sodium bicarbonate via the same access.

Alternatively Etoposide phosphate: Etoposide phosphate as etoposide-equivalent dose: 113 mg etoposide phosphate is equivalent to 100 mg etoposide. This difference is due to different molecular weights.

**Dacarbazine**

Caution: highly emetogenic! Reduce dose by 75% if GFR <60 ml/min or bilirubin >1.5 mg/dl. Avoid sun exposure due to photosensitivity.

**Dexamethasone**

Caution: Diabetics (no glucose infusions, blood sugar measurement on treatment days daily at 17:00)

**Literature**

Borchmann et al., Lancet 2024. <https://doi.org/10.1016/S0140-6736(24)01315-1>

**Appendix 2: Supplemental material**

**Figure S2: CONSORT chart**

**Received 3-Day BrECADD for cHL (N = 41)**

University Hospital of Cologne

10 Jan 2024 to 17 Jul 2026

**Included in Analysis (N = 30)**

**Excluded (N = 11)**

- Switched treatment site, incomplete data (N = 5)
- Started on 4-day schedule (N = 2)
- Salvage therapy, not first-line (N = 2)
- Prior anti-PD1 therapy (N = 1)
- Lost to follow-up during therapy (N = 1)

Abbreviations: BrECADD = brentuximab vedotin, etoposide, cyclophosphamide, doxorubicin, dacarbazine, and dexamethasone; cHL = classic Hodgkin lymphoma; PD1 = Programmed Cell Death Protein 1
